# Supplementary material for: Sevelamer Use and Mortality in People with Chronic Kidney Disease Stages 4 and 5 Not on Dialysis
Source: J Clin Med. 2023 Dec 12;12(24):7631. doi: 10.3390/jcm12247631 (PMC10743559; doi:10.3390/jcm12247631)
Supplement: Supplementary file 1 [file jcm-12-07631-s001.zip › jcm-2691534-supplementary.pdf]

Supplemental Material

Table S1. Hazard Ratios (HR) and 95% confidence intervals (CI) for the risk of all-cause and cardiovascular mortality, comparing Calcium-based binders-Treated Vs Untreated patients, unadjusted and after multivariable adjustments for various confounders.

		All-cause mortality		Cardiovascular mortality	
**Model No.**	**Covariates**	**HR (95% CI)**	***P***	**HR (95% CI)**	***P***
0	Unadjusted	1.01 (0.79-1.53)	0.6	1.26 (0.79-2.01)	0.3
1	Demographic, anthropometric characteristics and co-morbidities[a]	1.33 (0.92-1.90)	0.1	1.79 (1.06-3.03)	0.03
2	Model 1 plus medications[b]	1.11 (0.76-1.62)	0.6	1.56 (0.93-2.61)	0.1
3 (full)	Model 2 plus laboratory parameters[c]	0.81 (0.52-1.26)	0.4	1.33 (0.73-2.42)	0.4

Note: The same covariates were included in all the full-adjusted analysis. [a]Model 1 sex, age, body mass index, waist circumference, diabetes, cardiovascular comorbidity, and systolic and diastolic pressure. [b]Model 2 included all previous variables plus treatment with antihypertensive drugs, iron, erythropoiesis stimulating agents and both native and active vitamin D. [c]Model 3 (full model) was adjusted for those variables included in Model 1 and 2 plus serum levels of phosphate, iPTH, calcium, albumin, eGFR, hemoglobin, potassium, C-reactive protein and potassium, and 24h-proteinuria.
